# High-dose colistin pharmacokinetics in critically ill patients receiving continuous renal replacement therapy

**DOI:** 10.1186/s13613-024-01384-1

**Published:** 2024-09-28

**Authors:** Gennaro De Pascale, Lucia Lisi, Salvatore Lucio Cutuli, Carlotta Marinozzi, Altea Palladini, Elena Sancho Ferrando, Eloisa Sofia Tanzarella, Gianmarco Lombardi, Domenico Luca Grieco, Alessandro Caroli, Rikardo Xhemalaj, Laura Cascarano, Gabriella Maria Pia Ciotti, Claudio Sandroni, Maurizio Sanguinetti, Pierluigi Navarra, Massimo Antonelli

**Affiliations:** 1 Dipartimento di Scienze Biotecnologiche di Base, Cliniche Intensivologiche e Perioperatorie, Università Cattolica del Sacro Cuore, Rome, Italy; 2 Dipartimento di Scienze dell’Emergenza, Anestesiologiche e della Rianimazione, Fondazione Policlinico Universitario A. Gemelli IRCCS, Cattolica del Sacro Cuore Largo A. Gemelli 8, Rome, 00168 Italy; 3 Sezione di Farmacologia, Dipartimento di Sicurezza e Bioetica, Università Cattolica del Sacro Cuore, Rome, Italy; 4 Medical Intensive Care Unit, Hospital Clinic Barcelona, Barcelona, Spain; 5 Dipartimento di Scienze di Laboratorio e Infettivologiche, Fondazione Policlinico Universitario A. Gemelli IRCCS, Rome, Italy

**Keywords:** Sepsis, Infection, Colistin, Colistimethate, Critical care

## Abstract

**Background:**

Colistin, administered as intravenous colistimethate (CMS), is still used in the critical care setting and current guidelines recommend high dosage CMS in patients undergoing continuous renal replacement therapy (CRRT). Due to the paucity of real-life data, we aimed to describe colistin pharmacokinetic/pharmacodynamic (PK/PD) profile in a cohort of critically ill patients with infections due to carbapenem-resistant (CR) bacteria undergoing CRRT.

**Results:**

All consecutive patients admitted to three Intensive Care Units (ICUs) of a large metropolitan University Hospital, treated with colistin for at least 48 h at the dosage of 6.75 MUI q12, after 9 MIU loading dose, and undergoing CRRT were included. After the seventh dose, patients underwent blood serial sampling during a time frame of 24 h. We included 20 patients, who had CR-*Acinetobacter baumannii* ventilator-associated pneumonia and were characterized by a median SAPS II and SOFA score of 41 [34.5–59.3] and 9 [6.7–11], respectively. Fifteen patients died during ICU stay and six recovered renal function. Median peak and trough colistin concentrations were 16.6 mcg/mL [14.8–20.6] and 3.9 mcg/mL [3.3–4.4], respectively. Median area under the time–concentration curve (AUC_0 − 24_) and average steady-state concentration (C_ss, avg_) were 193.9 mcg h/mL [170.6–208.6] and 8.07 mcg/mL [7.1–8.7]. Probability of target attainment of colistin pharmacodynamics according to the *f*AUC_0 − 24_/MIC target ≥ 12 was 100% for MIC ≤ 2 mcg/mL and 85% for MIC = 4 mcg/ML, although exceeding the toxicity limit of C_ss, avg_ 3–4 mcg/mL.

**Conclusions:**

In critically ill patients with CR infections undergoing CRRT, recommended CMS dosage resulted in colistin plasmatic levels above bacterial MIC_90_, but exceeding the safety C_ss, avg_. limit.

**Trial registration:**

This trial was registered in ClinicalTrials.gov on 23/07/2021 with the ID NCT04995133 (https//clinicaltrials.gov/study/NCT04995133).

## Background

Carbapenem-resistant (CR) gram-negative bacteria are leading causes of hospital-acquired infections [1–4], being *Klebsiella pneumoniae*, *Pseudomonas aeruginosa* and *Acinetobacter baumannii* the most common species associated with worse clinical outcomes and representing a worldwide research priority for the development of newly effective molecules [5, 6]. Despite the recent introduction of new potent, well-tolerated, molecules against the above strains, old drugs, like polymixins, have still a clinical placement, like in the empirical phase, as part of combination strategy or as salvage therapy after first-line agents failure [7]. Colistin (polymixin E) is the most commonly used molecule in this class, being administered in the form of its prodrug, colistimethate (CMS), which is either hydrolysed to colistin or metabolised via the renal route [8]. Colistin is an amphiphilic antibiotic with homogeneous distribution within the extracellular body water and exerts a rapid bactericidal activity by linking to the lipopolysaccarides and disrupting the outer membrane of Gram Negative bacteria [8]. Unfortunately, it is burdened by a narrow therapeutic window due to its dose-dependent nephrotoxicity and neurotoxicity [9–12]. In patients with creatinine clearance (CrCl) ≥ 90 ml/min, a loading dose of 9 million IU of CMS followed by ≈10.9 million IU as maintenance daily dose is recommended to achieve a plasma average level at the steady state (C_ss, avg_) of 2 mcg/mL [13]. Little is known about colistin pharmacokinetics (PK) and pharmacodynamics (PD) during continuous renal replacement therapy (CRRT) [14, 15] and current guidelines [13], based on limited evidence, recommend increasing the maintenance CMS dose to ≈ 13.3 million IU/day [16]. However, this dosing strategy has never been prospectively assessed in a real-life setting. Indeed, we conducted an open-label, prospective study aimed to analyse the colistin PK/PD profile in critically ill patients undergoing CRRT.

## Methods

### Patients and study design

This was a prospective, open-label, observational study performed between 2021 and 2022 in three intensive Care Units (ICUs) (total 62 beds) of a 1500-bed teaching hospital in Rome, Italy. The study was conducted in accordance with the Declaration of Helsinki and the protocol was approved by the Catholic University’s Ethical Committee (EUDRACT 201-001019-95 ID-Study 3946). Written informed consent was obtained from the patients’ legally authorized representative. This manuscript was written according to the principles of “Strengthening the Reporting of Observational Studies in Epidemiology” (STROBE) [17].

All patients undergoing antibiotic treatment with high dose CMS as an empirical or targeted therapeutic strategy during continuous renal replacement therapy were evaluated for inclusion. Inclusion criteria were: age > 18 years, duration of treatment planned > 48 h, documented or suspected infection with a CR bacteria, stage 3 Acute Kidney Injury (AKI) according to the KDIGO classification [18], half-life of the CRRT filter < 48 h. Pregnant women, and patients with high probability of short-term death according to the simplified acute physiology score (SAPS II) were excluded.

According to current guidelines [13], CMS high dosage (HD) was administered intravenously (IV) at loading dose (LD) of 9 MIU mg over 30-min, followed by 6.75 MIU over 30-min q12. On day 4, at steady state, pharmacokinetic analyses of the study group were performed. CRRT was delivered as continuous veno-venous hemodiafiltration (CVVHDF) with Prismaflex ST150 (Gambro AB, Lund, Sweden) using the AN69 ST membrane (acrylonitrile-sodium-methyl sulfonate surface treated; surface area, 1.5m2) and regional citrate anticoagulation. Dose intensity was prescribed according to current guidelines [18], and with a ratio of dialysate to replacement fluid of 1:1. The CRRT circuit and membrane were changed every 72 h, as standard operating procedure in our center.

Clinical and demographic data were recorded upon enrolment. Sepsis was managed according to the Surviving Sepsis Campaign 2021 guidelines [19], Acute Respiratory Distress Syndrome (ARDS) was diagnosed according to the Berlin definition [20]. Renal recovery was defined as a reduction in peak AKI-KDIGO stage [21] and was evaluated in patients without chronic kidney disease.

### Sample collection and PK/PD analysis

Although colistin concentration-time profiles are stable just on day 3, blood samples were collected after the seventh dose (on day 4 of treatment) at T0 (immediately before the initiation of CMS infusion) and 1 h, 2 h, 4 h, 6 h, 8 h, 10 h and 12 h from the start of CMS infusion. First order kinetic determined pharmacokinetic parameters; maximum and minimum concentrations (*C*_max_, *C*_min_) were directly obtained from observed peak and trough concentrations. The 0–12 h area under the time–concentration curve (AUC_0–12_ ) was determined by the linear trapezoidal rule. Colistin AUC_0–24_ was calculated as AUC_0–12_ × 2. The average steady-state plasma concentration (*C*_*ss, avg*_) was obtained from the ratio of colistin AUC _0–24_ above T (i.e. the dosage interval). In all patients, distribution volume (*Vd*), drug clearance (CL), and elimination half-life (t_1/2_ ) were calculated after a single 6.75 MIU intravenous dose at steady state. According to current guidelines [13, 22], the recommended PK/PD therapeutic target has been considered the area under the plasma concentration- time curve across 24 h at steady state above MIC (*f*AUC_ss 0−24_ hr) of ~ 12 mg_hour/L, that equates to a target *C*_ss, avg_of ~ 2 mg/L of the total drug against susceptible strains (MIC ≤ 2 mcg/mL). Higher concentrations were shown to increase both the incidence and severity of AKI [13]. Colistin and CMS concentration were measured using and modifying the Gobin assay [23].

### Microbiological analysis

Bacterial isolates were identified by using matrix-assisted laser desorption ionization-time of flight mass spectrometry (MALDI-TOF MS). MICs were determined by a Micronaut AST system-based BMD, VITEK 2 AST-N397 card, according to the manufacturer’s instructions. EUCAST (version 11.0, 2021) clinical breakpoints were used to interpret MICs. In pneumonia episodes, microbiological evidence of bacterial infection was achieved by processing respiratory samples both for conventional and fast-microbiology panel testing. Gram staining results showed the presence of inflammatory cells in all samples, indicating that their collection had been performed appropriately. Semiquantitative culture results were obtained using calibrated loops to inoculate and streak samples on both selective/differential [24].

### Statistical analysis

All statistical analyses were performed using SPSS Statistical Software version 28.01.0 (IBM, Armonk, NY, USA), whereas data were graphed using GraphPad Prism version 6.0 (GraphPad Software, San Diego, CA, USA). Kolmogorov–Smirnov test was used to value the variables distribution. The data with a non-Normal distribution were assessed with Mann–Whitney test and the median and selected centiles’ (25th–75th) value were given (interquartile range, IQR). Categorical variables are presented as proportions. Due to the PK/PD design of the study, a sample size was not calculated, foreseeing the recruitment of 20 patients during the study period.

## Results

The clinical characteristics of the 20 enrolled patients are reported in Table 1. Median SAPS II score was 41 and the most relevant comorbidities were chronic heart disease, chronic obstructive respiratory disease, chronic renal failure and diabetes. All included patients were affected by CR-*Acinetobacter baumannii* ventilator associated pneumonia (VAP). Most patients needed invasive mechanical ventilation due to ARDS and received high dose vasopressors due to septic shock. The total median duration of mechanical ventilation, vasopressors and CRRT were 26 days, 6.5 days and 12.5 days, respectively. Five (75%) patients died, and 35% of survivors had renal recovery.

**Table 1:** Clinical characteristic of 20 patients enrolled

Baseline characteristics	
Age, years	70 [65.3–75.3]
Gender, male	16 (80)
BMI	30.5 [27-32.9]
SAPS II	41 [34.5–59.3]
Charlson Comorbidity Index	4.5 [2–5]
CHD	6 (30)
COPD	7 (35)
Diabetes	8 (40)
CKD	4 (20)
Immunosuppression	1 (5)
**Presenting Features & CRRT**	
VAP	20 (100)
Concomitant bacteremia	6 (30)
Septic Shock	13(80)
ARDS	18 (90)
SOFA	9 [6.7–11]
BAL *Acinetobacter* spp. isolation	20 (100)
Colisitn MIC mcg/mL	1[1-1.5]
Serum albumin, g/dL	2.1 [2-2.3]
Fluid balance, mL	+ 651.5 [323–797.5]
Hematocrit, %	28 [27–29]
Residual diuresis, mL/24 h	0
CVVHDF dose intensity, mL/kg/h	32.5 [30–35]
CRRT duration, days	12.5 [10–23.25]
**Outcome measures & Therapy**	
MV duration, days	26 [21.25–37.25]
Vasopressors duration, days	6.5 [4.5-11.25]
Death in ICU	15 (75)
Treatment failure	14 (70)
Renal Recovery*	6 (37.5)
Nebulized colistin therapy	18 (90)
Intravenous colistimethate therapy, days	13 [9.25-12]

Categorical variables are expressed in count (percentage); continuous variables are expressed as median [interquartile range]

*Abbreviations*: BMI: body mass index; VAP: Ventilator Associated Pneumonia; SAPS II: Simplified Acute Physiology Score II; CHD: Chronic Heart Disease; COPD: Chronic Obstructive Pulmonary Disease; CKD: Chronic Kidney Disease; MV: mechanical ventilation; ICU: Intensive Care Unit; ARDS: Acute Respiratory Distress Syndrome; SOFA: Sequential Organ Failure Assessment; BAL: bronchoalveolar lavage; BSI: Blood Stream Infection; MIC: Minimal Inhibitory Concentration; CRRT: continuous renal replacement therapy; CVVHDF: continuous veno-venous haemodiafiltration

*The rate of renal recovery was computed from the 16 patients without chronic renal failure

Concomitant bacteraemia was detected in 30% of the cases and the median duration of CMS treatment was 13 days [9.25-12]. All patients received a combination therapy with either cefiderocol (2 g q8–6 h), tigecycline (100 mg of q12 after a 200 mg loading dose), fosfomycin (8 g q8) or ampicillin-sulbactam (9 g q8); 18 out of 20 received concomitant nebulized CMS (5 MIU q8) [24]. The median CVVHDF dose was 32.5 ml/kg/h, and all patients were anuric; on the sampling day, median cumulative fluid balance, serum albumin and hematocrit were + 651.5 mL, 2.1 g/dL and 28%, respectively.

**Fig. 1:**
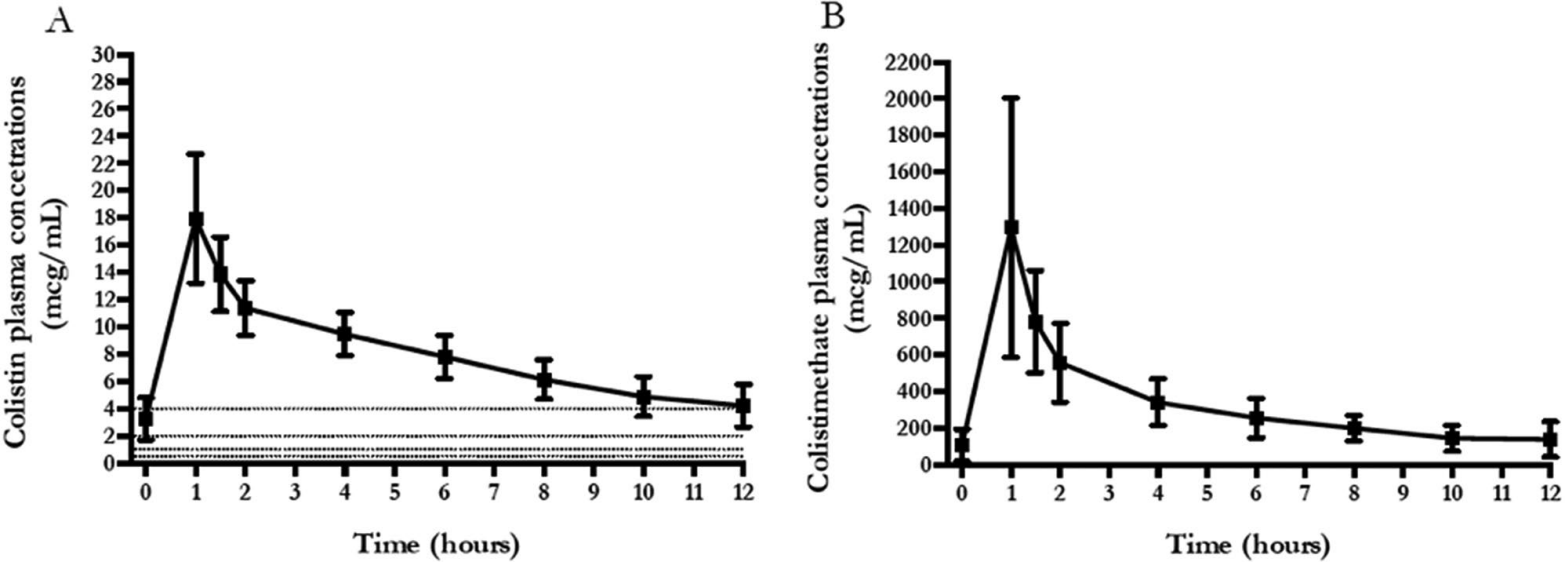
Time concentration curves of colistin (**A**) and colistimethate (**B**) in 20 patients enrolled

**Fig. 2:**
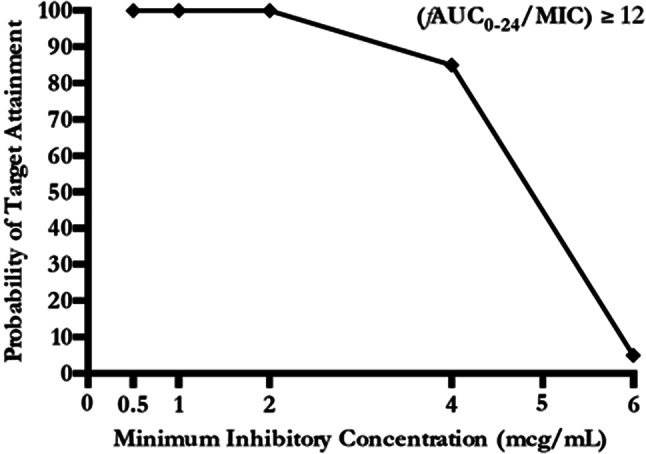
Probability of target attainment according to different MIC values

An one-compartment model with first-order disposition processes adequately described colistin concentration-time curve, although significant interindividual variability was observed. Median *Vd*, t_1/2_ and Cl_TOT_ were 126.37 L, 20.89 h and 2.08 L/h, respectively (Table 2). Median [IQR] values of C_max_, C_min_ and C_ss, avg_ were 16.65 [14.77–20.64] mcg/mL, 3.93 [3.29–4.37] mcg/mL and 8.07 [7.1–8.69] mcg/mL, respectively. Furthermore, C_ss, avg_ of colistin was significantly lower in patients who recovered renal function compared to the ones that had no renal recovery (7.48 [6.35, 8.05] vs. 8.31 [7.35, 9.13] mcg/mL, respectively; *p* = 0.026), while no differences were observed for C_min_, C_max_, and according to patient survival. Figure 1 shows the mean ± SD time–concentration profile at different time points of plasma CMS and colistin concentrations, compared with most frequently observed MIC values (0.5–1 – 2–4). Median [IQR] values of AUC_0 − 24_ and *f*AUC_0 − 24_ were 193.86 [170.6–208.65] mcg h/mL and 58.16 mcg h/mL [51.18–62.6], respectively. The probability of target attainment of colistin pharmacodynamics according to the *f*AUC_0 − 24_/MIC target of 12, according to increasing MIC values, is shown in Fig. 2. This dosing schedule allowed to attain the PD target (*f*AUC_0 − 24_/MIC ≥ 12) for 100% of isolates with MIC = 2 and 85% with MIC = 4 mcg/mL, although exceeding the toxicity limit of C_ss, avg_ 3–4 mcg/mL.

**Table 2:** Colistin PK/PD profile and CRTT settings

Colistin PK/PD parameters	
*C*_max_, mcg/mL	16.65 [14.77–20.64]
*C*_min_, mcg/mL	3.93 [3.29–4.37]
*C*_ss, avg_, mcg/mL	8.07 [7.1–8.69]
t_1/2_, h	20.89 [14.45–22.84]
*Vd*, L	126.37 [79.57-139.17]
CL_TOT_, L/h	2.08 [1.94–2.37]
CL_CRRT_, L/h	1.21 [0.88–1.35]
%CL_TOT_, L/h	49.2 [39.25–64.2]
*%E*	17.65 [14.98–21.67]
AUC_0 − 24,_ mcg h/mL	193.86 [170.6–208.65]
*f*AUC_0 − 24,_ mcg h/mL	58.16 [51.18–62.6]
*f*AUC_0 − 24_/0.5 mcg/mL ≥ 12	20 (1)
*f*AUC_0 − 24_/1 mcg/mL ≥ 12	20 (1)
*f*AUC_0 − 24_/2 mcg/mL ≥ 12	20 (1)
*f*AUC_0 − 24_/4 mcg/mL ≥ 12	17 (85)
*f*AUC_0 − 24_/6 mcg/mL ≥ 12	1 (5)

Categorical variables are expressed as count (percentage); continuous variables are expressed as median [interquartile range]

*Measure on the sampling day

*Abbreviations*: CMS: colistimethate; *C*_ssavg_: average steady-state concentration;. *C*_max_: maximum concentration; *C*_min_: minimum concentration; AUC: Area Under The Curve; *Vd*: distribution volume; t_1/2_: half-life; CL: clearance; *%E*: % filter extraction

## Discussion

Our study showed that high-dose CMS (9 MIU as loading dose, followed by 6.75 MUI q12) in critically ill patients with CR-*Acinteobacter baumannii* VAP undergoing CVVHDF resulted in higher than recommended C_ss, avg_ for germs with MIC ≤ 2 mcg/mL, although allowing to attain the PD target (*f*AUC_0 − 24_/MIC ≥ 12) in all treated patients.

Several variables may influence colistin plasma concentration as demographic (e.g. body mass), clinical (e.g. kidney function) and therapeutic (e.g. RRT modality, membrane, mode and dose) variables [25]. In order to account for CRRT removal of colistin, the 2019 international guidelines for the optimal use of polymyxins [13] recommend administering a daily dose of 440 colistin base activity (CBA), corresponding to ≈ 13,3 MIU or ≈ 1173 mg of CMS in patients who are exposed to this extracorporeal organ support. This recommendation derives from PK data indicating the need of a 10% increase in daily dose per 1 h of CRRT, with the aim of obtaining the target C_ss, avg_ 2 mcg/mL [13, 26]. In patients with renal failure, plasma concentrations of CMS, undergoing a considerable removal during CVVHDF with a significant lower conversion to the active drug, are higher than those of formed colistin. Additionally colistin has an extensive carrier-mediated tubular reabsorption in the kidney, which is not achievable with extracorporeal device currently used in clinical practice. The need for optimizing colistin use in such patients was identified after the first reports of drug under-exposure when using drug dosages according to the residual renal function [27, 28]. A subsequent study on five patients [29], used higher dosages of CMS until 2 MIU q8, showing suboptimal C_ss, avg_ (≤ 1 mcg/mL), a *f*AUC_0 − 24_/MIC lower than the effective PD target, a membrane extraction ratio of 68% and a CL_HDF_ above 4 L/h. Similarly, another PK/PD analysis on three patients confirmed high removal of colistin during CRRT, ranging between 45% and 60%, with the effluent clearance accounting for about 40% [30].

Interestingly, more recently, when CMS 4.5 MIU q12 were administered to eight critically ill patients receiving CRRT [31], median C_max_ values for CMS and colistin were 12.6 mg/L and 1.72 mg/L, respectively. The authors, documenting a 62% Cl_CVVHDF_, formulated a pharmacokinetic model where a LD of 12 MIU followed by a maintenance dosage of 6.75–7.5 MIU q12 would be needed to achieve a target C_ss, avg_ of 2–3 mcg/mL [31]. On top of that, in 2017 *Nation and co-workers* developed a guidance algorithm for colistin use in critically ill patients and indicated that a daily CMS dose of about 13 MIU daily would allow to achieve 80% of target attainment rate (C_ss, avg_ ≥ 2 mcg/mL) with less than 30% of risk to overcome the safety limit of C_ss, avg_ ≥ 4 mcg/mL [15].

Said that, some authors, when using high CMS daily administration schedules during CRRT, observed steady-state concentrations above 10 mcg/mL, especially in presence of residual renal function and when high-dose CMS was administered for a prolonged course [32–34]. Further, in the latest and largest investigation on this topic, ten critically ill patients requiring CRRT and receiving CMS 3 MIU q8 were prospectively studied [35]. The authors found that CRRT clearance accounted for 41% an 28% of total CMS and colistin clearance, being significantly influenced by haematocrit levels, and documented therapeutic colistin values of both AUC_0 − 24_ (569 [404–741] mcg h/mL) and C_ss, avg_ (4.43 [3.01–7.24] mcg/mL), although on the upper limit of the safety target.

The observed literature heterogeneity may have several explanations. First the proposed algorithm for high CMS daily dosage derives from a small population pharmacokinetic analysis on 29 patients including different RRT modalities, intensity, and residual renal function [14, 15]. Moreover, a risk of dosage confusion exists between CMS reported as IU or mg, and formed colistin reported as mg of CBA, without the correct universal conversion of CMS 1 MIU : CBA 30 mg : CMS 80 mg [22]. Moreover, the real unbound colistin fraction, which is variable according to the elevation of its major carrier, the alpha- 1-acid glycoprotein, may present a huge inter-patient variability, being responsible of the observed unpredictable pharmacokinetic profile [36].

In our study, all patients underwent CVVHDF with a similar daily dose and since this technique uses diffusion across a concentration gradient (e.g. hemodialysis) in addition to hemofiltration across a pressure gradient, the equivalences of the two modes with respect to CMS/colistin clearance are unknown. Additionally none of our patients had residual renal function and almost all were also receiving high dose nebulized CMS (5 MIU q8), delivered through vibrating mesh nebulizers, potentially contributing to the higher than expected steady-state colistin levels [24]. In our study we cannot ascribe the observed high C_ss, avg_ to a partial tubal reabsorption, but we used in all the patients the novel AN69 ST membrane, which allows much higher clearance through elimination at both membrane surface (adsorption, rapidly saturated) and in its bulk (transmembrane removal, less easily saturated). In addition, we cannot exclude a partial plasmatic absorption after CMS nebulization although recent results from PK studies documented colistin concentrations < 1 mcg/mL after aerosolization of either 3 MIU and 5 MIU CMS [37, 38].

In a recent post-hoc analysis of a large PK/PD study [12] the authors observed that for colistin Css, avg between 2.25 and 6.75 mcg/mL more than the majority of patients had ≥ 50% loss in baseline creatinine clearance and a value of 3 mcg/mL has been identified as the maximal tolerability threshold for Css, avg. Although no data are available on renal toxicity of high colistin concentrations in patients undergoing CRRT, in our population six (37.5%) out of the 16 patients without chronic renal disease in our population had renal recovery, stimulating a research question about colistin potential effects on the ability of patients to recover from sepsis-induced AKI.

We showed that the recommended CMS dosage resulted in high and potentially toxic colistin levels in patients undergoing CRRT. In our study, C_ss, avg_ of colistin was significantly lower in patients that had renal recovery compared to the ones that did not recover renal function, although this is an observational finding and does not imply causation. In contrast, the observed concentrations could be useful for less susceptible bacteria with an MIC = 4 mcg/mL or difficult-to-treat infections (i.e. pneumonia), in the initial empirical therapy phase, pending the susceptibility profile of the new and less toxic molecules. Moreover, the availability of colistin therapeutic drug monitoring could potentially be of help reducing the risk of achieving toxic colistin levels.

Our study has several limitations. First, we measured only total colistin and derived the unbound fraction from the available literature data. Second almost all the patients were receiving concomitant nebulized CMS as part of *A.baumanni* VAP treatment, whose adsorption fraction cannot be measured. Third we did not evaluate the potential neuro-toxicity of the observed colistin concentrations, since all the patients were deeply sedated and most were administered muscle relaxants [39, 40]. Fourth, we did not collect ultrafiltrate samples and volume, as well as pre-filter and post-filter blood samples, that would have been useful to evaluate the total (adsorption and transmembrane removal) colistin clearance [14] of the AN69-ST membrane.

Finally, we cannot draw any conclusion on CMS high dosage clinical value, not having any control group. However this is the largest study where colistin PK/PD has been investigated in patients receiving CRRT and where guidelines-recommended CMS dosage (≈ 13.5 MIU/day) has been prospectively evaluated in the critically ill setting.

## Conclusions

The use of recommended high dose colistimethate in critically ill patients undergoing CRRT resulted in steady-state average concentrations above colistin MIC_90_ of commonly isolated bacteria, but exceeding the recommended safety threshold. Apart from the early phase of empirical therapy, lower CMS daily dosages should be adopted in the clinical practice, especially when colistin therapeutic drug monitoring or alternative drugs are not available.

## Data Availability

The datasets used and/or analyzed during the current study are available from the corresponding author on reasonable request.

## Abbreviations

AKI: Acute Kidney Injury
ARDS: Acute Respiratory Distress Syndrome
CL: Clearance
AUC: Area under the time–concentration curve
CFU: Colony forming unit
Css, avg: Average concentration at the steady state
Cmax: Maximum concentration
Cmin: Minimum concentration
CR: Carbapenem-resistant
CMS: Colistimethate
CBA: Colistin base activity
CRRT: Continuous renal replacement therapy
CVVHDF: Continuous veno-venous hemodiafiltration
CrCl: Creatinine clearance
t1/2: Elimination half-life
IV: Intravenous
ICU: Intensive care unit
LD: Loading dose
MIC: Minimum inhibitory concentration
PD: Pharmacodynamics
PK: Pharmacokinetics
SAPS II: Simplified Acute Physiology Score II
TD: Toxicodynamic
VAP: Ventilator-associated pneumonia
Vd: Distribution volume

## References

[1] Vincent JL, Sakr Y, Singer M, Martin-Loeches I, Machado FR, Marshall JC, et al. Prevalence and outcomes of infection among patients in Intensive Care Units in 2017. JAMA. 2020;323(15):1478–87. 32207816 10.1001/jama.2020.2717 PMC7093816

[2] Tabah A, Buetti N, Staiquly Q, Ruckly S, Akova M, Aslan AT, et al. Epidemiology and outcomes of hospital-acquired bloodstream infections in intensive care unit patients: the EUROBACT-2 international cohort study. Intensive Care Med. 2023;49(2):178–90. 36764959 10.1007/s00134-022-06944-2 PMC9916499

[3] Vincent JL, Rello J, Marshall J, Silva E, Anzueto A, Martin CD, et al. International study of the prevalence and outcomes of infection in intensive care units. JAMA. 2009;302(21):2323–9. 19952319 10.1001/jama.2009.1754

[4] Vincent JL, Bihari DJ, Suter PM, Bruining HA, White J, Nicolas-Chanoin MH, et al. The prevalence of nosocomial infection in intensive care units in Europe. Results of the European prevalence of infection in Intensive Care (EPIC) study. EPIC International Advisory Committee. JAMA. 1995;274(8):639–44. 7637145

[5] Lombardi G, Tanzarella ES, Cutuli SL, De Pascale G. Treatment of severe infections caused by ESBL or carbapenemases-producing Enterobacteriaceae. Med Intensiva (Engl Ed). 2023;47(1):34–44. 36202744 10.1016/j.medine.2022.09.002

[6] Rando E, Cutuli SL, Sangiorgi F, Tanzarella ES, Giovannenze F, De Angelis G, et al. Cefiderocol-containing regimens for the treatment of carbapenem-resistant A. Baumannii ventilator-associated pneumonia: a propensity-weighted cohort study. JAC Antimicrob Resist. 2023;5(4):dlad085. 37484029 10.1093/jacamr/dlad085 PMC10359102

[7] Lim LM, Ly N, Anderson D, Yang JC, Macander L, Jarkowski A 3, et al. Resurgence of colistin: a review of resistance, toxicity, pharmacodynamics, and dosing. Pharmacotherapy. 2010;30(12):1279–91. 21114395 10.1592/phco.30.12.1279 PMC4410713

[8] De Pascale GA. How to use Colistin in the ICU. Clin Pulm Med. 2015;22(3):141–7.

[9] Pogue JM, Lee J, Marchaim D, Yee V, Zhao JJ, Chopra T, et al. Incidence of and risk factors for colistin-associated nephrotoxicity in a large academic health system. Clin Infect Dis. 2011;53(9):879–84. 21900484 10.1093/cid/cir611

[10] Sorli L, Luque S, Grau S, Berenguer N, Segura C, Montero MM, et al. Trough colistin plasma level is an independent risk factor for nephrotoxicity: a prospective observational cohort study. BMC Infect Dis. 2013;13:380. 23957376 10.1186/1471-2334-13-380 PMC3765824

[11] Horcajada JP, Sorli L, Luque S, Benito N, Segura C, Campillo N, et al. Validation of a colistin plasma concentration breakpoint as a predictor of nephrotoxicity in patients treated with colistin methanesulfonate. Int J Antimicrob Agents. 2016;48(6):725–7. 28128096 10.1016/j.ijantimicag.2016.08.020

[12] Forrest A, Garonzik SM, Thamlikitkul V, Giamarellos-Bourboulis EJ, Paterson DL, Li J et al. Pharmacokinetic/Toxicodynamic Analysis of Colistin-Associated Acute Kidney Injury in critically ill patients. Antimicrob Agents Chemother. 2017;61(11). 10.1128/AAC.01367-17 PMC5655114 28893780

[13] Tsuji BT, Pogue JM, Zavascki AP, Paul M, Daikos GL, Forrest A, et al. International Consensus guidelines for the optimal use of the polymyxins: endorsed by the American College of Clinical Pharmacy (ACCP), European Society of Clinical Microbiology and Infectious diseases (ESCMID), Infectious Diseases Society of America (IDSA), International Society for anti-infective pharmacology (ISAP), society of critical Care Medicine (SCCM), and Society of Infectious diseases pharmacists (SIDP). Pharmacotherapy. 2019;39(1):10–39. 30710469 10.1002/phar.2209 PMC7437259

[14] Garonzik SM, Li J, Thamlikitkul V, Paterson DL, Shoham S, Jacob J, et al. Population pharmacokinetics of colistin methanesulfonate and formed colistin in critically ill patients from a multicenter study provide dosing suggestions for various categories of patients. Antimicrob Agents Chemother. 2011;55(7):3284–94. 21555763 10.1128/AAC.01733-10 PMC3122440

[15] Nation RL, Garonzik SM, Thamlikitkul V, Giamarellos-Bourboulis EJ, Forrest A, Paterson DL, et al. Dosing guidance for intravenous colistin in critically-ill patients. Clin Infect Dis. 2017;64(5):565–71. 28011614 10.1093/cid/ciw839 PMC5850520

[16] Cutuli SL, Cascarano L, Lazzaro P, Tanzarella ES, Pintaudi G, Grieco DL et al. Antimicrobial exposure in critically ill patients with Sepsis-Associated Multi-organ Dysfunction requiring extracorporeal organ support: a narrative review. Microorganisms. 2023;11(2). 10.3390/microorganisms11020473 PMC9965524 36838438

[17] von Elm E, Altman DG, Egger M, Pocock SJ, Gotzsche PC, Vandenbroucke JP, et al. The strengthening the reporting of Observational studies in Epidemiology (STROBE) statement: guidelines for reporting observational studies. J Clin Epidemiol. 2008;61(4):344–9. 18313558 10.1016/j.jclinepi.2007.11.008

[18] Group KAW. KDIGO clinical practice guideline for acute kidney injury. Kidney Int Suppl. 2012;17:1–138.

[19] Evans L, Rhodes A, Alhazzani W, Antonelli M, Coopersmith CM, French C, et al. Surviving sepsis campaign: international guidelines for management of sepsis and septic shock 2021. Intensive Care Med. 2021;47(11):1181–247. 34599691 10.1007/s00134-021-06506-y PMC8486643

[20] Force ADT, Ranieri VM, Rubenfeld GD, Thompson BT, Ferguson ND, Caldwell E, et al. Acute respiratory distress syndrome: the Berlin definition. JAMA. 2012;307(23):2526–33. 22797452 10.1001/jama.2012.5669

[21] Chawla LS, Bellomo R, Bihorac A, Goldstein SL, Siew ED, Bagshaw SM, et al. Acute kidney disease and renal recovery: consensus report of the Acute Disease Quality Initiative (ADQI) 16 workgroup. Nat Rev Nephrol. 2017;13(4):241–57. 28239173 10.1038/nrneph.2017.2

[22] Roberts JA, Abdul-Aziz MH, Lipman J, Mouton JW, Vinks AA, Felton TW, et al. Individualised antibiotic dosing for patients who are critically ill: challenges and potential solutions. Lancet Infect Dis. 2014;14(6):498–509. 24768475 10.1016/S1473-3099(14)70036-2 PMC4181663

[23] Gobin P, Lemaitre F, Marchand S, Couet W, Olivier JC. Assay of colistin and colistin methanesulfonate in plasma and urine by liquid chromatography-tandem mass spectrometry. Antimicrob Agents Chemother. 2010;54(5):1941–8. 20176909 10.1128/AAC.01367-09 PMC2863609

[24] De Pascale G, Pintaudi G, Lisi L, De Maio F, Cutuli SL, Tanzarella ES et al. Use of High-Dose Nebulized Colistimethate in patients with colistin-only susceptible Acinetobacter baumannii VAP: clinical, pharmacokinetic and Microbiome features. Antibiot (Basel). 2023;12(1). 10.3390/antibiotics12010125 PMC9855104 36671325

[25] Gregoire N, Aranzana-Climent V, Magreault S, Marchand S, Couet W. Clinical pharmacokinetics and pharmacodynamics of Colistin. Clin Pharmacokinet. 2017;56(12):1441–60. 28550595 10.1007/s40262-017-0561-1

[26] Nation RL, Li J, Cars O, Couet W, Dudley MN, Kaye KS, et al. Framework for optimisation of the clinical use of colistin and polymyxin B: the Prato polymyxin consensus. Lancet Infect Dis. 2015;15(2):225–34. 25459221 10.1016/S1473-3099(14)70850-3

[27] Honore PM, Jacobs R, Lochy S, De Waele E, Van Gorp V, De Regt J, et al. Acute respiratory muscle weakness and apnea in a critically ill patient induced by colistin neurotoxicity: key potential role of hemoadsorption elimination during continuous venovenous hemofiltration. Int J Nephrol Renovasc Dis. 2013;6:107–11. 23776390 10.2147/IJNRD.S42791 PMC3681400

[28] Li J, Rayner CR, Nation RL, Deans R, Boots R, Widdecombe N, et al. Pharmacokinetics of colistin methanesulfonate and colistin in a critically ill patient receiving continuous venovenous hemodiafiltration. Antimicrob Agents Chemother. 2005;49(11):4814–5. 16251342 10.1128/AAC.49.11.4814-4815.2005 PMC1280168

[29] Karvanen M, Plachouras D, Friberg LE, Paramythiotou E, Papadomichelakis E, Karaiskos I, et al. Colistin methanesulfonate and colistin pharmacokinetics in critically ill patients receiving continuous venovenous hemodiafiltration. Antimicrob Agents Chemother. 2013;57(1):668–71. 23147733 10.1128/AAC.00985-12 PMC3535942

[30] Markou N, Fousteri M, Markantonis SL, Zidianakis B, Hroni D, Boutzouka E, et al. Colistin pharmacokinetics in intensive care unit patients on continuous venovenous haemodiafiltration: an observational study. J Antimicrob Chemother. 2012;67(10):2459–62. 22790220 10.1093/jac/dks257

[31] Karaiskos I, Friberg LE, Galani L, Ioannidis K, Katsouda E, Athanassa Z, et al. Challenge for higher colistin dosage in critically ill patients receiving continuous venovenous haemodiafiltration. Int J Antimicrob Agents. 2016;48(3):337–41. 27474468 10.1016/j.ijantimicag.2016.06.008

[32] Menna P, Salvatorelli E, Mattei A, Cappiello D, Minotti G, Carassiti M. Modified Colistin Regimen for critically ill patients with Acute Renal impairment and continuous renal replacement therapy. Chemotherapy. 2018;63(1):35–8. 29334366 10.1159/000484974

[33] Mariano F, Leporati M, Carignano P, Stella M, Vincenti M, Biancone L. Efficient removal of colistin A and B in critically ill patients undergoing CVVHDF and sorbent technologies. J Nephrol. 2015;28(5):623–31. 25249467 10.1007/s40620-014-0143-3

[34] Akers KS, Rowan MP, Niece KL, Stewart IJ, Mende K, Cota JM, et al. Colistin pharmacokinetics in burn patients during continuous venovenous hemofiltration. Antimicrob Agents Chemother. 2015;59(1):46–52. 25313211 10.1128/AAC.03783-14 PMC4291386

[35] Leuppi-Taegtmeyer AB, Decosterd L, Osthoff M, Mueller NJ, Buclin T, Corti N. Multicenter Population Pharmacokinetic Study of Colistimethate Sodium and Colistin dosed as in normal renal function in patients on continuous renal replacement therapy. Antimicrob Agents Chemother. 2019;63(2). 10.1128/AAC.01957-18 PMC6355613 30478168

[36] De Pascale G, Sandroni C, Antonelli M. Colistin use in critically ill patients: in search of the optimal dosing. Chest. 2011;139(1):234. author reply – 5. 21208893 10.1378/chest.10-2031

[37] Gkoufa A, Sou T, Karaiskos I, Routsi C, Lin YW, Psichogiou M, et al. Pulmonary and systemic pharmacokinetics of colistin methanesulfonate (CMS) and formed colistin following nebulisation of CMS among patients with ventilator-associated pneumonia. Int J Antimicrob Agents. 2022;59(6):106588. 35405269 10.1016/j.ijantimicag.2022.106588

[38] Boisson M, Jacobs M, Gregoire N, Gobin P, Marchand S, Couet W, et al. Comparison of intrapulmonary and systemic pharmacokinetics of colistin methanesulfonate (CMS) and colistin after aerosol delivery and intravenous administration of CMS in critically ill patients. Antimicrob Agents Chemother. 2014;58(12):7331–9. 25267660 10.1128/AAC.03510-14 PMC4249558

[39] Madia F, Merico B, Primiano G, Cutuli SL, De Pascale G, Servidei S. Acute myopathic quadriplegia in patients with COVID-19 in the intensive care unit. Neurology. 2020;95(11):492–4. 32601119 10.1212/WNL.0000000000010280

[40] Luigetti M, Iorio R, Bentivoglio AR, Tricoli L, Riso V, Marotta J, et al. Assessment of neurological manifestations in hospitalized patients with COVID-19. Eur J Neurol. 2020;27(11):2322–8. 32681611 10.1111/ene.14444 PMC7405467

